# The Number of Louse Eggs on Wild Japanese Macaques (*Macaca fuscata*) Varies with Age, but Not with Sex or Season

**DOI:** 10.1007/s10764-017-9998-1

**Published:** 2017-11-10

**Authors:** Naomi Ishii, Takuya Kato, Taiki Uno, Ichirou Tanaka, Hiroshi Kajigaya, Shin-ichi Hayama

**Affiliations:** 1 Nippon Veterinary and Life Science University, Musashino, Tokyo 180-8602 Japan; 2 Yokkaichi University, Yokkaichi, Mie 512-8512 Japan

**Keywords:** Ectoparasites, Grooming, Host age, Host sex, Seasonality

## Abstract

During grooming, primates remove harmful ectoparasites, such as ticks and lice, and there is direct evidence for a health benefit of tick removal. Grooming behaviors differ among primates with respect to age and sex. Moreover, the number of ectoparasite may exhibit seasonal variation. Therefore the number of ectoparasites on a host may vary with effects, host age and sex, and season. However, these effects have not been a focus of louse infestation studies of primates. Grooming in Japanese macaques is related to sex and age, with developmental changes in behavior corresponding to the timing of tooth eruption. Moreover, behavioral data for Japanese macaques suggest that lice load may differ with the season. Thus, we examined whether the number of louse eggs varies according to host macaque sex, age, and season, and whether it changes in response to tooth eruption. We counted unhatched and hatched eggs attached to the hair on six 1-cm^2^ areas on the left wrist skin of culled macaques, using a stereoscopic microscope. We sampled five winter coats and three summer coats for each age class: infant, juvenile, adolescent, and adult. The number of unhatched and hatched eggs was related to age, but not to sex and season. There were significant differences in the number of unhatched eggs between infants and adults, juveniles and adults, and adolescents and adults. There were also significant differences in the number of hatched eggs between infants and adults, juveniles and adults, adolescents and adults. Tooth eruption did not influence the number of louse eggs. These results suggest that researchers should consider the age of host animals when assessing the relationship between grooming and ectoparasites.

## Introduction

Grooming is a major defense strategy against ectoparasites, and is performed by various species, including insects, fishes, birds, and mammals (Sparks 1967). For example, cleaning symbioses are particularly well documented in marine ecosystems (Cote 2000; Johnson *et al*. 2010). In insects, the Asian honey bee (*Apis cerana*) shows self-cleaning behavior when inoculated with mites (*Varroa jacobsoni*: Peng *et al*. 1987). Animals groom using various methods and body parts, including the mouth, finger, and beak, and researchers have suggested that ectoparasite removal during grooming has a direct effect on well-being in a variety of species, e.g., cats (*Felis domestica*: Eckstein and Hart 2000), antelopes (Hart 2000), meerkats (*Suricata suricatta*: Kutsukake and Clutton-Brock (2006), and fishes (Johnson *et al*. 2010).

Primates also remove harmful ectoparasites, such as ticks and lice, as well as louse eggs, during grooming, e.g., Japanese macaques (*Macaca fuscata*: Tanaka and Takefushi 1993), chimpanzees (*Pan troglodytes*: Zamma 2002b), and baboons (*Papio cynocephalus*: Akinyi *et al*. 2013). Ticks and lice transmit protozoa and bacteria, which cause serious infections, such as babesiosis (Kjemtrup and Conrad 2000; Ruebush *et al*. 1981), epidemic typhus (Blanc and Woodward 1945; Burgess 1995; Roux and Raoult 1999), trench fever (Roux and Raoult 1999), and relapsing fever (Burgess 1995; Durden 2001). Moreover, they suck the blood of host animals and cause anemia in humans (Guss *et al*. 2011; Speare *et al*. 2006) and a wide range of wild mammals (Durden 2001), including spider monkeys (*Ateles*: Ronald and Wagner 1973). In light of the negative health effects that result from tick and lice infestation, removal of ectoparasites by grooming may play a significant role in maintaining host health.

Female nonhuman primates groom more than males (Minami 1987; Mitchell and Tokunaga 1976). Differences in grooming may lead to differences in the number of ectoparasites and health. This hypothesis is supported in the case of ticks: baboons that receive more grooming have fewer ticks (Akinyi *et al*. 2013). The authors suggested that grooming protects baboons from the detrimental effects of ticks because individuals with more ticks suffer from health effects, such as skin wounds and low packed cell volume, an indicator of anemia. Allogrooming rates and the occurrence of lice and nits vary among groups of red howlers (*Aloutta seniculus*: Sánchez-Villagra *et al*. 1998), and studies of Japanese macaques have shown that grooming site preferences correlate with the distribution of louse eggs (Zamma 2002a) and that grooming may limit louse burden (Duboscq *et al*. 2016).

Sex-related differences in grooming appear during development (Minami 1987). Hair density also influences the number of louse eggs (Zamma 2002a) and decreases with age (Inagaki and Hamada 1985; Inagaki 1992). For example, age-related differences in behavior and hair length lead to differences in head lice in humans (Borges and Mendes 2002; Speare and Buettner 1999). Thus, differences in host age may also lead to differences in the number of ectoparasites in other primates.

The number of ectoparasites may also exhibit seasonal variation. For example, seasonal fluctuations in hormones (Nozaki 1991) associated with seasonal breeding of the host animals may influence reproduction of *Phthiraptera* (Duboscq *et al*. 2016; Durden 2001; Foster 1969). However, no reports have evaluated seasonal changes in the number of ectoparasites of primates directly.

Grooming in Japanese macaques is closely connected to louse eggs. Almost all (98.9%) of the objects that macaques pick up and then eat during grooming are louse eggs (*Pedicinus obtusus* and *P. eurygaster*) (Tanaka and Takefushi 1993), and the potential for feeding on louse eggs may motivate grooming behavior (Onishi *et al*. 2013). Japanese macaques also remove ticks, e.g., *Haemaphysalis longicornis*, during grooming (Tanaka and Takefushi 1993; Zamma 2002a), but this is rarely observed and the number of ticks on Japanese macaques is much smaller than that of lice (Zamma 2002a).

As in other primates, e.g., pigtail macaques (*Macaca nemestrina*) and bonnet macaques (*Macaca radiate*: Defler 1978), baboons (Young *et al*. 1982), red howlers (Sánchez-Villagra *et al*. 1998), Japanese macaques exhibit sex- and age-dependent differences in grooming behavior. Females groom other individuals significantly more frequently than males from about 28 weeks of age, and females continue to increase their rate of grooming until age 1 yr. (Eaton *et al*. 1985). Moreover, grooming between unrelated macaques occurs mainly among females (Ando 1982). Mothers preferentially groom their youngest offspring (Koyama 1977) and adolescent males spend slightly more time self-grooming than other age–sex classes (Maruhashi 1981). Changes in technique for effectively removing louse eggs are related to the ontogenetic growth of incisors (Tanaka and Takefushi 1993). Two types of louse egg handling techniques have been reported in Japanese macaques: removal using the lower incisors and removal using the first finger and thumb (Tanaka and Takefushi 1993). Although juveniles with lacteal incisors appear to be able to remove lice eggs accurately, egg removal becomes less accurate when the permanent incisors erupt. In contrast, adult macaques appear to use mainly their first fingers and thumbs to remove louse eggs (Tanaka and Takefushi 1993). Additionally, behavioral data indirectly suggest that lice load on Japanese macaques is higher in summer and fall than in spring and winter (Duboscq *et al*. 2016). The authors suggested that seasonal change of louse habitat caused by seasonal fluctuations in hormones and seasonal changes of hair density and length of host macaques may cause seasonal differences of lice load (Duboscq *et al*. 2016).

We examined sex-, age-, and season-related differences in the number of louse eggs on the fur of free-ranging Japanese macaques. We took advantage of a planned cull of Japanese macaques to measure the number of louse eggs on different individuals. Lice spend their entire lifecycle on their host (Wall and Shearer 2001) and female lice glue their eggs onto the hair shafts of their mammalian hosts (Durden 2001), meaning that we can count louse eggs on dead host macaques because they are attached to the hair (Tanaka and Takefushi 1993). Based on known variation in grooming behavior among Japanese macaques as well as known seasonal differences in hair density, we hypothesized that the number of louse eggs found on fur, i.e., those that were not removed by grooming, depends on the sex and age of the host and season. We tested three predictions: 1) females that are groomed more frequently by other individuals than are males have fewer eggs than males do; 2) age classes differ in the number of eggs, owing to differences in the amount of grooming they receive; 3) the number of louse eggs differs between individuals that have a winter coat and those that have a summer coat.

## Methods

### Data Collection

We counted the number of louse eggs on the carcasses of 64 wild Japanese macaques inhabiting Fukushima City, Fukushima Prefecture, Japan. At the request of Fukushima City, licensed hunters culled macaques from June 2009 to July 2011. The Fukushima Mirai Agricultural Cooperative froze macaque carcasses in Fukushima City, and we then transported the frozen carcasses to the Nippon Veterinary and Life Science University, where we defrosted carcasses at room temperature for 2 days to 1 week. We adjusted the defrosting period depending on the season to avoid putrefaction of the carcasses. After defrosting, we dissected the carcasses and collected skin samples.

The number of louse eggs varies between body parts, and many louse eggs are found on the arm (Zamma 2002a), where it is relatively easy to collect skin samples. Moreover, louse eggs are more often found on the outer side of the body than on its inner side (Zamma 2002a). To avoid bias in louse egg counts, we obtained all skin samples from within 5 cm from the root of the left wrist of each macaque, and scanned both the inner and outer side isometrically.

During necropsy, we determined the sex and estimated the age of each macaque using tooth eruption status in accordance with Iwamoto *et al*. (1987). We divided individuals into the following groups based on estimated age: infant (<1.5 yr), juvenile (1.5–3.5 yr), adolescent (3.5–5.5 yr), and adult (≥5.5 yr). We also noted whether or not juvenile macaques had adult incisors. After necropsy and sampling skin, we froze all samples for processing at a later date.

To assess seasonal differences in the number of louse eggs, we categorized individuals into those with a winter coat (culled between October and March) and those with a summer coat (culled between April and September) following the methods of Inagaki and Nigi (1988). We sampled five winter coats and three summer coats for each age–sex class (Table I).

**Table I:** Sample size of Japanese macaques at Fukushima City, Fukushima Prefecture, Japan, by age, sex, and season (June 2009–July 2011)

Age	Sex	Season	*N*
Infant	Male	Winter	5
		Summer	3
	Female	Winter	5
		Summer	3
Juvenile^a^	Male	Winter	5
		Summer	3
	Female	Winter	5
		Summer	3
Adolescent	Male	Winter	5
		Summer	3
	Female	Winter	5
		Summer	3
Adult	Male	Winter	5
		Summer	3
	Female	Winter	5
		Summer	3

^a^11 of 16 individuals had permanent incisors, and the other 5 individuals had lacteal incisors only

We defrosted skin samples at room temperature and counted the number of louse eggs. We randomly selected three 1-cm^2^ areas of skin from both the outer and inner side of the left wrist. In total, we sampled six areas for each macaque. We examined hairs on each of the six areas using a stereomicroscope (Shimazu STZ-40TBITb, Tokyo, Japan). If objects were attached to the hair, we cut the hair at the root, fixed it, and stored it in acetone. The same person processed all skin samples and extracted all objects.

### Data Analysis

We assigned objects collected from skin samples to one of three categories: unhatched eggs, hatched eggs, or other objects. Two observers sorted all objects using a stereoscopic microscope to reduce any bias in the sorting of small objects. The degree of concordance between the results of the two observers was high (κ = 0.86). We therefore used the results from just one observer for statistical tests.

We used a univariate negative binomial regression analysis for each variable to assess the associations of sex, season, and age of monkeys with the total counts of unhatched and hatched eggs remaining on hair shafts close to the host skin on all skin patches for each individual. We then used *post hoc* pairwise comparisons to compare age classes with Bonferroni adjustment for multiple comparisons after univariate negative binomial regression analysis for unhatched and hatched eggs. We used Stata/IC 13.1 (StataCorp LP, College Station, TX, USA) for analysis.

We used Student’s *t*-tests in R. 3.2.3 (R Core Team 2015) to test the difference in numbers of unhatched and hatched eggs between juveniles with lacteal incisors and those with permanent incisors.

## Ethical Note

Licensed hunters set box traps and shot all macaques used in this study at the request of Fukushima City between June 2009 and September 2011 as a measure against crop damage. Fukushima Prefecture made both the decision to cull animals and the decision of which animals to cull independently of this study. We did not influence these decisions. Licensed hunters captured and killed monkeys with the permission of the governor of Fukushima Prefecture, Japan, according to the Fukushima Japanese Monkey Management Plan, which was established based on the Wildlife Protection and Hunting Management Law of Japan. The method of culling was in accordance with the guidelines published by the Primate Research Institute, Kyoto University.

## Results

In total, we detected 2036 objects in samples from 45 of the 64 macaques, and no objects on the remaining 19 individuals. We counted 294 unhatched eggs, 1631 hatched eggs, and 111 other objects.

**Fig. 1:**
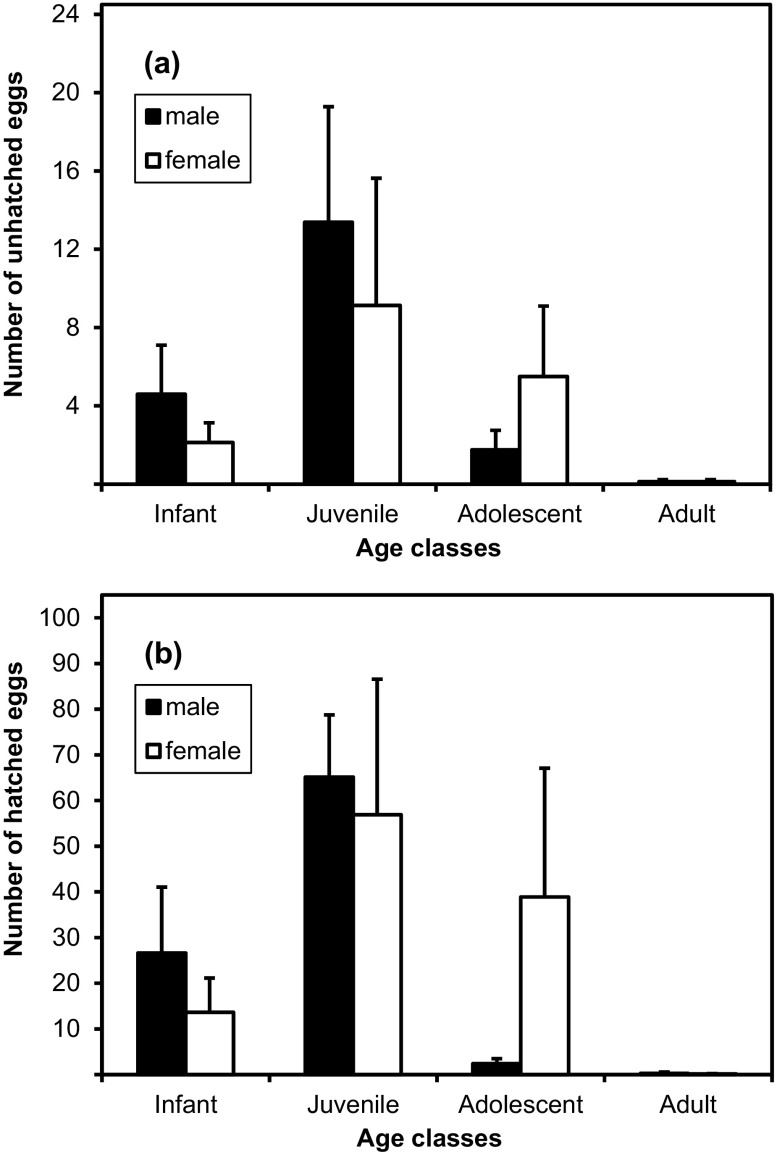
Mean and standard error of the number of **(a)** unhatched and **(b)** hatched eggs for each sex and age class of Japanese macaques at Fukushima City, Fukushima Prefecture, Japan (June 2009–July 2011)

Age had an effect on the number of the unhatched and hatched eggs, but sex and season had no effect on the number of the unhatched and hatched eggs (Tables II and III). Pairwise comparisons showed that the number of unhatched eggs differed significantly between infants and adults, juveniles and adults, and adolescents and adults (Table III, Fig. 1). The number of hatched eggs differed significantly between infants and adults, juveniles and adults, and adolescents and adults (Table III, Fig. 1).

**Table II:** Results of univariate negative binomial regression analysis testing for factors associated with the number of unhatched and hatched eggs on Japanese monkeys at Fukushima City, Fukushima Prefecture, Japan (June 2009–July 2011)

			Coefficient	95% CI	*P*-value
Unhatched eggs	Sex (*N* = 64)		0.16	−8.70 to 1.20	0.757
	Season (*N* = 64)		−0.85	−1.90 to 0.19	0.108
	Age	Infant (*N* = 16)	Reference		
		Juvenile (*N* = 16)	1.20	0.06 to 2.35	
		Adolescent (*N* = 16)	0.07	−1.10 to 1.24	
		Adult (*N* = 16)	−3.30	−5.09 to −1.50	
Hatched eggs	Sex (*N* = 64)		−0.15	−1.22 to 0.93	0.786
	Season (*N* = 64)		0.17	−0.94 to 1.28	0.767
	Age	Infant (*N* = 16)	Reference		
		Juvenile (*N* = 16)	1.11	−0.04 to 2.26	
		Adolescent (*N* = 16)	0.02	−1.13 to 1.18	
		Adult (*N* = 16)	−4.68	−6.29 to −3.07

**Table III:** Results for pairwise comparison with Bonferroni adjustment of hatched and unhatched eggs between each age group on Japanese monkeys at Fukushima City, Fukushima Prefecture, Japan (June 2009–July 2011)

	Age		Contrast	Standard error	*Z*	*P* > |*Z*|	95% Cl
Unhatched egg	Infant	Juvenile	1.20	0.59	2.06	0.239	0.56 to 2.35
		Adolescent	0.07	0.60	0.12	1.000	−1.10 to 1.24
		Adult	−3.30	0.92	−3.60	0.002	−5.09 to −1.50
	Juvenile	Adolescent	−1.13	0.58	−1.94	0.316	−2.28 to 0.01
		Adult	−4.50	0.91	−4.96	< 0.001	−6.28 to −2.72
	Adolescent	Adult	−3.37	0.91	−3.68	0.001	−5.16 to −1.58
Hatched egg	Infant	Juvenile	−5.28	0.98	1.89	0.349	−0.04 to 2.26
		Adolescent	−6.26	1.04	0.04	1.000	−1.13 to 1.18
		Adult	−7.24	1.10	−5.69	< 0.001	−6.29 to −3.07
	Juvenile	Adolescent	−8.23	1.17	−1.85	0.384	−2.23 to 0.06
		Adult	−9.21	1.23	−7.05	< 0.001	−7.39 to −4.18
	Adolescent	Adult	−10.19	1.30	−5.72	< 0.001	−6.31 to −3.09

The mean numbers of unhatched and hatched eggs did not differ between juvenile macaques that had lacteal incisors and those with permanent incisors (*t*-test: unhatched eggs, *t* = 1.17, df = 14, *P* = 0.26; hatched eggs, *t* = 1.08, df = 14, *P* = 0.30).

## Discussion

Our results indicate that the number of louse eggs on hair shafts close to the skin varies significantly among macaque age groups, but not by sex and season. There was a significant difference in unhatched eggs between infants and adults, juveniles and adults, and adolescents and adults. There was also a significant difference in hatched eggs between infants and adults, juveniles and adults, and adolescents and adults. Juveniles harbored more hatched and unhatched eggs than other age classes, but there was no significant difference in unhatched and hatched eggs between juveniles and adolescents. This may be due to one adolescent macaque that had 27 unhatched eggs and 234 hatched eggs, while all other adolescents had fewer than 15 unhatched eggs and fewer than 30 hatched eggs. It is unclear why this individual was such an outlier. There were no significant differences in the numbers of unhatched or hatched eggs between juvenile macaques with lacteal vs. permanent incisors. The number of louse eggs found on macaques might relate to factors such as grooming rates, hair density, and hormone levels. Moreover, host immunity and habitat might also influence the number of eggs. However, our results are generally consistent with those of previous studies where the number of unhatched and hatched eggs seemed to be affected by grooming (Duboscq *et al*. 2016; Zamma 2002a), and it is possible to explain some of the variability across age groups using known patterns of grooming behavior.

Grooming partners of Japanese macaques are nonrandom, and more than half of grooming occurs between relatives (Ando 1982; Koyama 1991; Nakamichi and Shizawa 2003; Oki and Maeda 1974). In particular, about 70% of all grooming between relatives occurs between mothers and their offspring (Ando 1982; Koyama 1991). However, the relationship between mothers and their offspring depends on the number of offspring, with the amount of grooming each offspring receives from the mother being negatively related to offspring number and the youngest offspring receiving more than a third of all mother–offspring grooming (Koyama 1977). The mean pregnancy rate among wild Japanese macaques aged ≥4 yr. living in Fukushima City was estimated to be 49% (Hayama *et al*. 2011), with females of reproductive age giving birth once every other year. Juveniles between 1.5 to 3.5 yr. old are likely therefore to have a younger sibling, with this younger sibling receiving more grooming from their mothers than they do. This difference in rates of maternal grooming may partly explain the observed difference in the number of louse eggs between infants and juveniles. Maternal grooming might be particularly important in preventing lice-borne infections in infants, since serum immunoglobulin content gradually rises with increasing age, up to sexual maturity (cynomolgus monkeys: Terao 2009).

Juveniles may struggle to remove louse eggs by themselves. Although we hypothesized that the number of louse eggs might differ between juveniles with lacteal vs. permanent incisors, we found no significant differences in the numbers of unhatched and hatched eggs between these two groups. Together with the high numbers of louse eggs on juveniles, this suggests that juvenile macaques may not be able to remove louse eggs accurately, irrespective of the grooming method used.

Although the number of lice living on the macaques is unclear because we did not count live adult lice, the observation that >80% of the 1925 louse eggs detected were hatched suggests that adult lice would have been present on the macaques before death. Zamma (2002a) suggests that it is difficult for monkeys to remove all lice by hand, and that both lice and louse eggs are expected to maintain a certain population level. We can infer that juvenile macaques, with the most hatched eggs, are parasitized by many adult lice. Lice pass from host to host most efficiently when “bridges” between hairs exist (Bowman 2009). Given that social play with direct contact in Japanese macaques occurs between individuals whose age disparity is <3 yr. (Imakawa 1990; Koyama 1985; Mori 1974), contact between juveniles is likely to be high, leading to cross-infections.

Sex- and/ or season-related differences have been reported in some cases of *Phthiraptera* infestation (Durden 2001; James *et al*. 1998; Murray 1963; Rassami and Soonwera 2012; Volf 1991); however, our results did not indicate sex- and season-related differences in the number of lice eggs on Japanese macaques. Lice load calculated indirectly by data on louse egg-picking gestures is higher in the summer and fall compared to the spring and winter (Duboscq *et al*. 2016). Our direct count data that found no seasonal difference in the number of louse eggs may reflect egg removal during grooming, which suggests prevention of season-related and potentially sex-related differences in infestation by grooming. However, we cannot definitively support this suggestion for two reasons. First, whereas Duboscq *et al*. (2016) divided the year into four seasons, in the present study we used just two seasons, which may mask changes in lice egg number over the year. Second, it is unclear whether the absence of sex differences is related to differences in removal behavior, or whether there were simply no sex differences to begin with. Further examination is needed to test our hypothesis, for example, using a combination of behavioral observations and direct counts of louse eggs.

We did not measure grooming behavior directly in this study, and behavioral studies are needed to conclude that grooming patterns are linked to age-related infection differences. Other potential causes include differences in hair density (Zamma 2002a). The density of Japanese macaque hair is not dependent on sex (Inagaki and Hamada 1985), but decreases during development (Inagaki 1992). A higher hair density on juveniles than on adults could explain the greater number of louse eggs observed in juveniles in our study, consistent with the results of Zamma (2002a). However, infants have even higher hair density than juveniles, yet appear to have fewer louse eggs, such that hair density alone cannot explain the observed differences in the number of louse eggs across the different age groups. Higher maternal grooming rates of infants may interact with hair density differences to create the observed patterns.

Host immunity may also play a role in lice infestation. In tick infestations, basophils play an essential and nonredundant role in antibody-mediated acquired immunity (Wada *et al*. 2010). Moreover, when a host acquires resistance against ticks, feeding by ticks is reduced (Wada *et al*. 2010). There are no studies to our knowledge that report how the Japanese macaque’s immune system reacts to lice. However, Durden (2001) suggested that systemic immunity to lice can probably develop in parasitized mammals, and that cellular responses presumably operate in wild mammals. Based on the host response to ticks (Wada *et al*. 2010), it is possible that louse infestations also cause immune reactions, and the number of lice and louse eggs may be higher on individuals that do not have fully functioning immune systems and cannot prevent pediculosis.

Finally, differences in environmental temperature may affect the number of louse eggs. Japanese macaques huddle in groups, and the size of these groups increases as the temperature drops, creating greater potential for interindividual transmission (Hanya *et al*. 2007; Takahashi 1997; Wada *et al*. 2007; Zhang and Watanabe 2007). Hair density is also related to temperature; macaques that live in cold regions have a higher hair density than macaques in warm areas, and may also have higher numbers of louse eggs (Inagaki 1992).

In conclusion, our results show that the number of louse eggs differs across individuals and with host age. Sex and/or age have not been a focus of many previous studies of the relationship between grooming and ectoparasites, but a difference in louse burden with host age may lead to differences in health. Researchers should examine primate grooming data, ectoparasite count data, and primate health data to test this hypothesis (as in Akinyi *et al*. 2013). Grooming in primates may have a social function (Boccia *et al*. 1989; Dunbar 1991; Schino *et al*. 1988) and/or a hygienic function (Barton 1985; Hutchins and Barash 1976; Reichard and Sommer 1994). There is evidence of decreased heart rate (Aureli *et al*. 1999; Boccia et al., 1989) and stress biomarkers (Shutt *et al*. 2007) and improved psychological well-being (Keverne *et al*. 1989) in relation to grooming. However, to our knowledge, only one report shows direct evidence for the hygienic function of primate grooming (Akinyi *et al*. 2013). Future studies should clarify the health benefits of ectoparasite removal, to further our understanding of the function of grooming.
